# Inclusive Education for Children with Developmental Disabilities in Ethiopia: Stakeholder Views on Benefits, Disadvantages and Priorities for Action

**DOI:** 10.1007/s10803-024-06549-2

**Published:** 2024-09-26

**Authors:** Olivia Burningham, Amanda Chen, Elisa Genovesi, Winini Belay, Ikram Ahmed, Moges Ayele, Fikirte Girma, Liya Tesfaye Lakew, Charlotte Hanlon, Rosa A. Hoekstra

**Affiliations:** 1 Department of International Development, London School of Economics and Political Science, London, UK; 2 St Andrew’s Mission School, Singapore, Singapore; 3 Department of Psychology, Institute of Psychiatry, Psychology & Neuroscience, King’s College London, London, UK; 4 Centre for Innovative Drug Development and Therapeutic Trials for Africa, Addis Ababa University, Addis Ababa, Ethiopia; 5 Department of Psychiatry, School of Medicine, College of Health Sciences, WHO Collaborating Centre for Mental Health Research and Capacity-Building, Addis Ababa University, Addis Ababa, Ethiopia; 6 School of Psychology, College of Education and Behavioral Studies, Addis Ababa University, Addis Ababa, Ethiopia; 7 Nia Foundation Joy Center for Autism, Addis Ababa, Ethiopia; 8 Centre for Clinical Brain Sciences, University of Edimburgh, Edimburgh, UK

**Keywords:** Inclusive education, Autism, Developmental disabilities, Ethiopia, Africa

## Abstract

Children with developmental disabilities (DD) including intellectual disability and autism, experience exclusion from social life and education in Ethiopia. Including children with DD in mainstream inclusive classes has potential to expand access to education and uphold their right to learn alongside typically developing peers. However, inadequate support in inclusive settings can hinder their participation and educational achievement. This study explores the perspectives of stakeholders on the ways in which inclusive education can support the needs or exacerbate the challenges of children with DD in Addis Ababa, Ethiopia, and on recommendations to address the challenges. Thirty-nine participants with expertise or experience relevant to children with DD, including caregivers, educators, clinicians and other experts, took part in semi-structured interviews. Their responses were analysed using thematic template analysis. The themes developed identify four aspects which are most relevant to the potential positive and negative consequences of inclusive education for children with DD: learning and development, peer relationships, safety in school and inclusion in society. The themes encompass sub-themes of positive and negative consequences, and suggested improvements to facilitate positive effects. Identified priorities for action include enhanced teacher training, awareness-raising initiatives, tailoring infrastructure and manpower to safeguard children with DD and promoting government focus on successful inclusion. These recommendations can be implemented to facilitate well-structured inclusive education, where children with DD are supported to participate alongside typically developing peers, as well as to safeguard against the potential negative consequences of inclusion, paying sufficient attention to the distinctive needs of children with DD.

Developmental disabilities (DD) are a group of conditions including autism, intellectual disability and ADHD, characterised by difficulties or delayed development in language, learning and/or behaviour (World Health Organization, 2013). Children with DD, especially those living in low- and middle-income countries (LMICs), are often excluded from mainstream services and do not receive the additional support they need compared to typically developing children, resulting in poorer outcomes across educational, health and psychosocial domains (UNICEF, 2021; WHO & UNICEF, 2023).

Children with DD may be educated in segregated settings apart from typically developing children, in inclusive settings together with them or in arrangements combining these two models. The practice of segregated special education is not without criticism, largely because it can be seen as perpetuating discrimination against these children through denying them the right to learn alongside other children as equals (Vergunst et al., 2021). This has produced an increasing focus on inclusive education internationally, including the Salamanca Statement and Framework for Action in 1994 (UNESCO, 1994), with the fundamental principle that all children should learn together (Ainscow et al., 2019). The Education 2030 Incheon Declaration and Framework for Action (UNESCO, 2015) emphasises the need for inclusion as the foundation for quality education (Ydo, 2020).

Despite the increasing promotion of inclusive education, tensions exist around the desirability of inclusive education in relation to its effects on children with DD. In its ideal conceptualisation, inclusive education has the potential to improve student outcomes on several dimensions, including access to education, acceptance by the school community, participation of students in activities, and achievement of students (Artiles et al., 2006), mirroring the goals specified in the Education 2030 Incheon Declaration (UNESCO, 2015). Quantitative evidence of the benefits of inclusive education is limited and focused on Western high-income countries and reports mixed results on whether inclusion in mainstream schools benefits academic achievement, socioemotional development and wellbeing of children with disabilities (including DD; Dalgaard et al., 2022). Case studies of inclusive education interventions in LMICs, such as from Guatemala and Malawi (Banks & Zurmond, 2015), have suggested their potential to improve access to education for children with disabilities through its provision in mainstream schools. However, they did not necessarily improve educational quality—observed in the acceptance and participation of children with disabilities within schools—due to constraints in material and personnel resources (Werning et al., 2016). The concern surrounding educational quality extends to Ethiopia, where inclusive education is perceived to benefit students with disabilities psychologically by offering a sense of belonging and empowerment, while failing to meet their educational needs to the same extent as segregated classrooms (Franck & Joshi, 2017). Among the challenges that hinder quality education provision in inclusive settings, limited teacher training and the inability to provide individualised support due to large class sizes, of typically 60–70 students (Beyene & Tizazu, 2010; Franck & Joshi, 2017; Ginja & Chen, 2021). It is unclear whether the situation may differ when focusing on children with DD specifically, rather than considering disability more broadly, including motor and sensory disabilities.

Across several African countries, substantial challenges exist in providing inclusive education for children with DD (Genovesi et al., 2022; Okyere et al., 2018). Compared to children with physical and sensory disabilities, the education of children with DD is often viewed with scepticism by policymakers and educators (Okyere et al., 2018). This may partly be due to the distinctive behavioural challenges and requirements for additional support manifested by children with DD. Another challenge is the common belief that children with DD are incapable of learning, such that efforts to include them in mainstream schools are perceived as a waste of resources (Okyere et al., 2018). Further barriers to including children with DD in mainstream schools in African countries are found in large class sizes (Drame & Kamphoff, 2014; Genovesi et al., 2022; Oswald & Swart, 2011), absent or inadequate teacher training, both pre-service and in-service, about the needs of children with DD, and a lack of human, material and financial resources to facilitate inclusion (Genovesi et al., 2022; Mangope et al., 2018; Okyere et al., 2018).

## Ethiopian Context

The majority of children with DD who come to clinical attention in Ethiopia have substantial support needs; most are non-speaking, have moderate to profound intellectual disability and many have additional health needs such as epilepsy. Due to lack of awareness and limited diagnostic services, families with children with symptoms of autism or ADHD without co-occurring intellectual disability are unlikely to seek help, although clinicians working in private practice report this pattern is slowly changing in Addis Ababa. There is a conspicuous lack of education services available for children with DD, both inclusive and segregated (Tekola et al., 2016). Indeed, as many as 74.5% of caregivers of children with DD attending government child mental health out-patient clinics reported unmet educational needs for their child (Tilahun et al., 2016). While some specialised centres in the Ethiopian capital Addis Ababa offer education for children with DD, they are highly limited in capacity and are often inaccessible to many families, particularly those living in rural areas (Tekola et al., 2016). With these centres being insufficient to accommodate all children with DD, many are unable to access any form of education (Tekola et al., 2016).

The Ethiopian Federal Ministry of Education has emphasised its commitment to providing education for children with disabilities across iterations of its *Education Sector Development Programme* (ESDP) since the programme’s third edition (Ministry of Education, 2005). However, the Ministry has not yet achieved the actual transformations within the education system necessary for inclusion (Franck & Joshi, 2017), and inclusive classrooms and schools remain scarce (Tefera et al., 2015). The Ministry’s sixth and latest ESDP (2021) estimated that only 11% of children with special needs were enrolled in primary education, decreasing to 2.8% for secondary education. While these statistics refer to children with any disability, the issue of lack of access to education in Ethiopia is likely exacerbated for children with DD compared to children with sensory or physical disabilities. The existing challenges of delivering educational or therapeutic services for children with DD in the low-resource setting of Ethiopia are compounded by pervasive stigma towards children with DD and their families (Tekola et al., 2020). Strategic plans of the government, such as those laid out in the *Master Plan for Special Needs Education/Inclusive Education 2016–2025* (Ministry of Education, 2016), also seem to convey a weaker focus on DD compared to sensory and physical disabilities, with specific recommendations for materials, training and infrastructure being tailored towards the latter.

Ethiopia’s commitment to inclusive education is affirmed in its ratification of the United Nations Convention on the Rights of Persons with Disabilities (CRPD) (United Nations, 2006). This stands in contrast to the current situation of limited acceptance and retention of children with DD in Ethiopia’s mainstream schools (Tekola et al., 2016).

## Aim

Before considering the potential for implementing inclusive education (Genovesi et al., 2023, 2024), it is necessary to build a shared understanding of the meaning, acceptability of, and motivations for, inclusive education, driven by the local community (Singal, 2006). This enables inclusive education to be seen as “reflective of, and creative of, inclusion in society”, rather than being imposed in an imperialistic manner (Thomas, 2013). In the present study, we seek to understand the perspectives of stakeholders on the ways in which inclusive education can support the needs, or instead exacerbate the challenges, of children with DD in Addis Ababa, Ethiopia. We also highlight improvements that stakeholders consider crucial to increase the potential benefit of inclusive education for children with DD. This in-depth analysis complements a further study, which, drawing from the same dataset, analyses contextual and implementation factors for including children with developmental disabilities in primary school settings in Addis Ababa. Genovesi et al. (2023, 2024) report two framework-driven overviews of context and implementation. The more conceptual analysis presented in this paper is necessary to ensure that any implementation plans are rooted in local understandings of their potential to improve the lives of children with DD.

## Methods

### Study Design

We explored stakeholders’ perspectives through a qualitative interview study. Our epistemological position was critical realism (Wiltshire, 2018) as we investigated the potential consequences of inclusive education for children with DD in specific contextual reality, while acknowledging that such context was explored through participants’ perspectives and influenced by the research team’s interpretations, each shaped by their personal experiences and knowledge.

### Context and Setting

The study was conducted in the capital city of Ethiopia, Addis Ababa. Addis Ababa has a population of over five million people (World Population Review, 2023), with Amharic as the dominant language. While more recent official data is unavailable, a survey in children reported that 87% of the primary school-age population of Addis Ababa and 36.3% of secondary school-aged children were schooled (Central Statistical Agency (CSA) [Ethiopia] & ICF, 2016). As previously reported (Tekola et al., 2016), there are a few special education centres for autistic children and children with intellectual disabilities within Addis Ababa (Tekola et al., 2016).

### Participants

Participants fell into several categories: seven clinicians; three government officials; three academics; four non-governmental organisation (NGO) representatives; thirteen professionals working at primary schools (public mainstream, private inclusive, private special), including eight teachers, two school heads or principals, three other members of staff responsible for psychological and education support programmes; and nine caregivers of a child with DD, both formally diagnosed and undiagnosed in-keeping with the study context of a lack of diagnoses. One clinician was also a caregiver of a child with DD, bringing the total to 10 caregivers.

We had planned to recruit 8–10 caregivers, 10–15 school representatives (including teachers and principals or managers) and 10–15 other professional informants (including NGO representatives, academics, clinicians) and government officials. These numbers were selected as they were anticipated to provide both variety and depth of perspectives on the topic, enabling in-depth analysis of the research question, and were met in our recruitment, as detailed above.

Teachers, principals and caregivers were approached at schools, and some caregivers were additionally recruited from clinics. Purposive sampling from different settings ensured that the recruitment of teachers and principals represented special, mainstream and inclusive educational backgrounds, and caregivers’ children with DD received different types of education, if any. Other informants with work expertise relevant to children with DD and their education were purposively selected based on publicly available information and prior collaborations of the research team. Snowballing was also used through informant suggestions and referrals, as local informants could suggest potential participants who were knowledgeable about the topic.

### Data Collection

In-depth, semi-structured interviews were conducted individually with all participants (*N* = 39). Duration of interviews ranged from 18 to 133 min. Interviews were conducted either virtually via video-call (*n* = 5) or face-to-face in Addis Ababa (*n* = 34). Eighteen interviews were conducted by UK-based researcher EG in English, and the remaining twenty-one were conducted in the local language of Amharic by Ethiopian co-investigators IA and WB. All interviews were audio-recorded and subsequently transcribed. All participants provided informed consent for participation and audio-recording of their interviews. Participants were offered a payment of around £4 in the local currency as reimbursement for their time.

The interviews were conducted using a topic guide, including questions regarding perspectives on the current provision of education for children with DD, perceptions of inclusive education and its outcomes, barriers and facilitators to inclusive education, and potential ways to promote inclusion for these children. Additional questions were tailored to each participant’s particular area of expertise, to maximise the information obtained from the stakeholders and utilise their differing perspectives (see the Appendix for topic guides). To ensure clarity of the questions, at the start of the interview, we reiterated our definition and examples of DD, also included in the information sheet used in the informed consent process. The definition included difficulties or delayed development in language, learning and/or behaviour (World Health Organization, 2013), particularly intellectual disabilities, autism and ADHD, but excluding specific learning disabilities such as dyslexia or dyscalculia.

A brief demographic survey was used to collect participants’ personal information, including their gender, age, education level and occupation. Caregiver participants answered additional questions on their family background and their child’s developmental information.

### Data Analysis

The 18 interviews conducted in English were transcribed by first authors AC and OB and the remaining 21 Amharic interviews were transcribed and translated to English by third-party professionals.

Data were analysed using template thematic analysis (Brooks et al., 2014), an approach that is highly flexible in relation to theme conceptualisation and development and lends itself well to group analysis. Four authors (OB, AC, EG and WB) coded all transcripts by tagging segments of data that could be assessed meaningfully in relation to inclusive education for children with DD. The initial codes were developed inductively, with discussions and iterations among coders, enabling reflexivity in the development of ideas and interpretations of particular phrases. All coding was conducted using the software NVivo, release 1.7.1. Following coding of all transcripts, the coding template was refined to improve clarity of codes and remove duplicate codes, jointly done by OB, AC and EG. Further input from the rest of the team, particularly Ethiopian experts (IA, MA, WB, FG, LT), clarified meanings of local idioms and phrases expressed in Amharic interviews and provided a contextual perspective on codes.

For this report, we developed in-depth themes answering the specific research question: “How can inclusive education support the needs or exacerbate the challenges of children with DD in Addis Ababa, and what can be done to enhance its benefits and reduce its disadvantages?” We typically identified themes at the semantic level, making interpretations based on the explicit meaning of data. However, we also considered latent themes representing ideas and assumptions underlying semantic content, in line with our philosophical stance of reality being explored through participants’ perspectives. We recognised the influence of past literature on inclusive education when developing themes, but strived to remain open-minded and derive themes primarily based on the data collected.

### Reflexivity

The research team comprised four UK-based investigators, one European investigator based in Ethiopia and five Ethiopian investigators with different areas of expertise. Team members are researchers with expertise in Global Mental Health, DD and inclusive education, students, clinicians, and a caregiver of a child with DD and special education practitioner. All members had a strong belief that increasing access to quality inclusive education for children with DD was a positive goal to strive towards within the Ethiopian context. Throughout the analysis, authors were mindful of their own positionality, and held team discussions in which they could share and reflect upon their interpretations of the data.

### Ethical Approval

The study was granted ethical approval from the Research Ethics Office at King’s College London (reference RESCM-22/23-21930), the Scientific and Ethical Review Committee at CDT-Africa (Addis Ababa University), and the Addis Ababa University College of Health Science Institutional Review Board (reference 061/21/CDT).

## Results

### Participant Demographics

The study comprised 39 (20 women) participants, aged from 28 to 65 years. Professional stakeholders had work experience ranging from 1 to 30 years. Eight caregivers were housewives, of whom 5 had attended 0–3 years of formal education, while 3 had completed secondary school; one was a trader and had completed primary school and one was a qualified and practicing clinician. Caregivers’ children (*n* = 10; 6 female) ranged in age from 8 to 24 years, with diagnoses including autism (7), cerebral palsy (1) and ADHD (2), often co-existing with intellectual disability (though sometimes only informally observed by teachers) and epilepsy.

Table 1 presents the thematic template resulting from the analysis. Each theme and subtheme is described below and exemplified by quotes of participants, identified with E—for interviews conducted in English—or A—for interviews in Amharic—and a unique number.

**Table 1:** Outline of theme development

	Main theme		Sub-themes
1	Learning and development of children with DD	1a	Benefit: Collaborative learning alongside typically developing children
		1b	Disadvantage: Limited support inhibiting learning
		1c	Priority for action: Improved teacher training – DD-specific – Competency-based – Practical – Regular
2	Peer relationships of children with DD	2a	Benefit: Building social relationships with typically developing peers
		2b	Disadvantage: Vulnerability of children with DD to bullying
		2c	Priority for action: Raising awareness of DD among typically developing children
3	Safety of children with DD in an inclusive school	3a	Disadvantage: Risk to safety of children with DD in inclusive schools
		3b	Priority for action: Safety considerations in infrastructure and manpower decisions
4	Societal inclusion of people with DD	4a	Benefit: Greater acceptance of people with DD in the community
		4b	Disadvantage: Poorly implemented inclusion reinforces stigma
		4c	Priority for action: School readiness for children with DD

### Theme 1: Learning and Development

### 1a. Benefit: Collaborative Learning Alongside Typically Developing Children

The opportunity for children with DD to learn from typically developing peers was viewed as a benefit of inclusive education. Firstly, participants frequently noted improvements in children’s social and communicative skills, following continual interaction with typically developing peers. Secondly, academic progress was reported, facilitated by peer support offered by typically developing children. One participant expressed satisfaction at her child’s academic performance on par with typically developing peers, which she attributed to his learning in an inclusive school:

the problem was with the language and social interaction, so that part improved more. And his educational status when you see is average performance. […] So his knowledge also is comparable with the other kids. If he was not enrolled in this inclusive school, he may miss that part also. (E14, caregiver)

When children are supported in their learning and development and allowed to participate on increasingly equal footing with typically developing peers, their self-esteem is built up—as teacher A27 explained: “I will not skip slowly developing children when they raise their hands to answer questions. This helps them develop confidence. No one laughs when they make mistakes.” Consultant E08 remarked that enabling children with DD “to feel that they get equal opportunity with the typical ones” was significant as it “would help [them] to have confidence, being similar with others”; similarly, teacher A23 summarised that “inclusion will make them feel that they are not inferior to others.”

Thirdly, participants reported reductions in challenging behaviour of children with DD following inclusive education. Learning alongside typically developing peers was seen to improve behavioural regulation of children with DD:

In inclusive education, if we facilitate for them, they can imitate the typical kids’ activities, roles and in everything. […] The teachers are not telling him what he’s doing, but he was imitating his peer, so he was doing better.

(E07, teacher)

This sentiment was echoed by a caregiver whose daughter attended a special school:

The good thing about including autistic children in regular classrooms is that autistic children learn by seeing. They learn from their non-disabled peers. My daughter would have learned many things from other children if she had joined [regular] classrooms. (A31, caregiver)

This perception of children’s propensity to learn by imitation, besides accounting for an advantage of inclusive education, also raises a drawback of segregated special education, in that children may copy behaviours from classmates with DD with greater support needs or harmful behaviours:

They were complaining that the other children were worse than him, so he couldn’t improve. That was the very first thing they say. Everybody, even when you ask them the reason that they didn’t enrol him in special needs school, yeah, they would say that that would worsen their symptoms rather than improve, when he sees other children with worse symptoms. (E13, clinician)

#### 1b. Disadvantage: Limited Support Inhibiting Learning

Participants reported that teachers in Ethiopia are strained in their capacity to manage students due to large class sizes. Coupled with their limited skill and knowledge on DD and inclusive education, this makes it difficult for them to adequately support children with disabilities: “in a class with the size of 60 or 70, […] how can that teacher teach all the 50 in a class and go to that student [with DD] to provide that support, is this feasible?” (E05, NGO representative). Therefore, teachers may “overlook [children with DD] so that they [the children] can just spend the day there at school, and not attain any meaningful learning sessions or education” (E18, NGO representative).

As students are taught with a curriculum and teaching methods that make no provisions for the needs of children with DD, children with DD may struggle to progress academically:

I don’t think it is a good idea. Do you know why? The slowly developing children will lag behind the non-disabled students. I think they should be taught separately according to their potential. I think it is unfitting for the children. (A25, caregiver)

The difficulty of children with DD to keep pace in mainstream academic environments, relative to their peers who progress more smoothly, can injure their morale and self-esteem: “Their feelings might get hurt if they can’t compete with their non-disabled peers [or] if they fail to do what their non-disabled peers can do” (A26, caregiver).

Children with DD may feel especially discouraged or humiliated if placed alongside younger children based on their learning ability. Caregiver A24’s daughter “started spending time with little kids” and “she said she is not a small kid and didn’t want to learn in the same class as them.”

The lack of support for the learning and development of children with DD in mainstream schools, which amplifies their learning difficulties relative to typically developing children, was thus a concern to participants. This lack of support was particularly stark in comparison to special schools:

The special schools are established specially for slowly developing children. […] They have trained professionals and have better infrastructure availability. Also, they have management preparedness and are dedicated to their work. Therefore, they use better training methodologies and treat the children in a better way. If you come to government-owned schools, the education is called inclusive in the absence of specialised support services. Honestly speaking, there is a huge difference between the [mainstream] schools and the special schools. (A19, academic)

This perception of the inadequacy of inclusive education in the government sector to support children with DD was reflected in some caregivers’ preference for their children to learn in special schools, such as caregiver A20 who “would like [her child] to be enrolled in the separate special needs schools because it is better than the regular schools. They will treat him better in the separate special needs schools.”

#### 1c. Priority for Action: Improved Teacher Training

Stakeholders considered the lack of appropriate training as a major cause of teachers’ inability to support children with DD in inclusive schools. Clinician E11 highlighted that mainstream teachers sometimes have not heard of DD, making inclusion challenging: “for example, the teacher [who] was teaching mathematics for that class, doesn’t really know what autism is, doesn’t really know how to include that child with autism in her class.” Based on stakeholders’ suggestions, we outline four key features highlighted by stakeholders that training to improve teachers’ support for children with DD should have: DD-specific, competency-based, practical and regular.

*DD-specific*. Firstly, in contrast to the training currently received by special needs teachers — “more inclined to other types of impairments, such as you know, visually impaired or hearing impaired, focusing on the Braille trainings or the sign language trainings, or things like that” (E18, NGO representative) — stakeholders called for DD-specific training. For example, according to teacher A22, “teachers should [be trained] to detect, categorise and help the different types of slowly developing children. Teachers need the training to do this.”

*Competency-based.* Secondly, stakeholders listed several specific competencies that teachers should be trained in, while current courses are “very general and just highlight about everything” and “that doesn’t help for intervention.” (E08, academic). Clinician E01 suggested that “they should be trained on how to manage difficult behaviour, how to engage children, how to, you know, be available emotionally or how to manage these children and how to attend [to] their educational needs.” Furthermore, the method in which teachers communicate with children with DD is important, as highlighted by teacher A21: “People might use words that affect the morale of children with special needs. When people get training in special needs education, they will know how to communicate with slowly developing children and treat them with love.” Thus, teachers across mainstream schools in Addis Ababa should be trained in multiple competencies to support the learning and development of children with DD.

*Practical.* Thirdly, stakeholders highlighted that teachers should receive practical training beyond learning theories regarding DD and inclusion, in light of the current dominance of theory-based education, as put by consultant E03: “Still majority, I can say more than 90% of the trainings are […] teaching them the same methodologies, the same you know, the same inclusion principles, not engaging them with the practical activities happening at school level.” Practical training can function to correct misperceptions of DD and negative attitudes rooted in a lack of first-hand experience with children with DD:

sometimes, some people expect when the door is opened, the kids are screaming or crying or shouting or showing some weird behaviour, but when they see and learning, it’s very different. […] So putting in the real environment will be better. (E07, teacher)

Beyond improving prospective teachers’ awareness of DD, practical training was deemed necessary for teachers to learn strategies to handle children with DD in classrooms. Gaps in these skills were remarked on by participants such as clinician E11: “they don’t know what to do when they are facing these kids”. Participants called for hands-on practice to be included in training for teachers to support children with DD in order to acquaint them with the characteristics of children with DD and impart practical skills to manage the children in classrooms.

*Regular.* Finally, participants highlighted that training for teachers should be offered regularly rather than once-off. Teacher E07 commented on the importance of “continuous training, not one time; [teachers] need refreshment [refresher] trainings”, sharing about his experience of his school’s regular sessions for staff to discuss optimal teaching methods. To make inclusive education a supportive environment for children with DD, the knowledge and skills of teachers across Addis Ababa with regards to supporting children with DD should be continually updated according to the latest evidence in the field.

### Theme 2: Peer Relationships of Children with DD

#### 2a. Benefit: Building Social Relationships with Typically-Developing Peers

Within inclusive education, children with DD have opportunities to socialise with their typically-developing peers during lessons and other school activities, such as excursions or festive celebrations. Besides supporting the academic and behavioural development of children with DD, as explained in Theme 1a, these interactions also promote social development and enable the children to build positive social relationships. While effort on the part of teachers was sometimes necessary to “train the children to be close to one another” (A35, teacher), genuine and tight-knit friendships could develop between children with and without DD:

They play games with them. Their relationship is so close that they miss one another when the school closes for winter recess. […] [Children with DD] don’t leave the classroom alone. They do everything their non-disabled peers do. (A35, teacher)

These friendships could be supportive in nature, with typically-developing children assisting children with DD to participate in activities, particularly if teachers “train non-disabled children to help children with special needs in the classroom”, as does teacher A35. Regardless of teachers’ involvement, peer-to-peer social interaction itself can enable typically-developing children to “know about their peers more and […] be more kind, more attentive to others” (E01, clinician), such that typically developing children can be mindful of the unique needs that their peers with DD may have.

The necessity of social connections for children with DD was underscored by teacher A27: “A child that comes to school and a child kept behind locked doors are not the same. A child locked behind closed doors might watch movies, but they will not experience social life.” Inclusive schools offer a further advantage of socialisation in a “surrounding whereby the society is, you know, resembled; everyone is there” (E18, NGO representative), which is more reflective of society than the silo of “a special and isolated school” (E15, clinician). Inclusive education can thus be “the ideal platform for [children with DD] to […] integrate in a society” (E18, NGO representative), as children can assimilate into social networks more smoothly following their education.

#### 2b. Disadvantage: Vulnerability of Children with DD to Bullying

Social isolation of children with DD in inclusive settings was a pervasive issue reported by participants. With their differences in cognition and behaviour, children with DD may be alienated by typically developing peers, or “be vulnerable to verbal and physical assault” (A32, caregiver):

In one class, because these guys really lack social skills, so they don’t communicate, they don’t fit in to that, so there is much more bullying, so they segregate themselves to the end of the class. (E16, school representative)

It was suggested that caregivers may be unwilling to enrol their child with DD in school due to fears of bullying: “in our country, slowly developing children face insult and bullying. Parents will be scared to send slowly developing children to schools because of that.” (A19, academic).

Participants acknowledged the psychologically damaging impact of these destructive social interactions on children with DD, with clinician E12 noting that children “would have low self-esteem and other kind of psychological distress or traumatic kind of situation may occur there with other children”. Caregiver A32 also reflected this concern: “Other children run away when they see them, laugh at them, and tease them. That could hurt their feelings.”

#### 2c. Priority for Action: Raising Awareness of DD Among Typically Developing Children

Stakeholders expressed that bullying typically arises from a lack of understanding about DD among typically developing children: “If the other kids will not understand the children with limitations, they will abuse them, bullying.” (E14, caregiver). To mitigate potential occurrences of bullying, stakeholders thus recommended educating typically developing children about DD so that they can better understand their peers. A caregiver raised the importance of building this awareness before children with DD are included into a school: “Regular classroom children will accept slowly developing children if you teach them about that before the inclusion is done… awareness should come first.” (A32, caregiver). This awareness should also incorporate an understanding of the different needs of children with DD compared to typically developing children: “if we try to create awareness about the different needs of these children in a more accepting environment, such kinds of things might decrease the occurrence of these unfortunate incidents.” (E01, clinician).

Some stakeholders also described previous efforts to create awareness among typically developing children—one teacher highlighted the effectiveness of awareness-raising in improving attitudes of typically developing children towards those with DD, and how children were then able to support their peers with DD within an inclusive setting:

They used to see them as strange creatures when we started inclusive education. That improved after we went around the classrooms and created awareness of slow mental development in children. We went to each classroom and told students to support their slowly developing classmates. They support them and make them participate in their group work. (A28, teacher)

Thus, raising awareness about DD among typically developing children can enable friendships to be forged across children of varying abilities, creating a supportive social environment in inclusive schools.

### Theme 3: Safety of Children with DD in an Inclusive School

#### 3a. Disadvantage: Risk to Safety of Children with DD in Inclusive Schools

Concerns regarding the physical safety of children with DD in inclusive schools were raised by participants. As well as being harmed by peer bullies, the risks of children fighting with other children, falling down or experiencing other forms of harm were identified by caregivers: “I also fear that he could attack people or hurt himself.” (A20, caregiver). These risks were also observed by a teacher in an inclusive school: “They might hit people. They might hit tables. […] The child assigned to me was over-active and unstable. He ran here and there. He didn’t know he could hurt himself if he fell.” (A35, teacher). These worries were compounded by limited supervision in inclusive schools, possibly caused by understaffing, with caregiver A20 expressing that “[n]o one watched [the children] when they fought with one another”. The lack of supervision also meant that children with DD could exit the school unsupervised and come to harm: “What if they enrol him in the school and he escapes their watch and gets hit by a car?” (A20, caregiver). One teacher reported an instance of harm to a child, further legitimising these concerns:

Yes, we get worried when they miss classes. We worry that they were hit by a car when they came to school. Our student got kidnapped once, and they brought him back after some days. Maybe they wanted to take money from his parents. We worry, as teachers and fathers when things like this happen. (A23, teacher)

Another important barrier to children’s wellbeing in inclusive education is the risk of sexual abuse against girls with DD: “I think that sexual abuse of children with an intellectual disability is a problem of great concern. Much needs to be done to address the issue.” (A32, caregiver). One stakeholder also expressed concerns that should a child with DD be assaulted, their impairment may mean that they are less able to report it: “The parent will feel that if she is a girl, if they send [her] to school, […] she couldn’t tell she is raped or she is, like she’s sexually abused.” (E05, NGO representative).

Stakeholders’ concerns for children with DD in this respect extended beyond the school compound to children’s journeys to and from school. For instance, caregiver A24 expressed regarding her daughter: “She can’t go far away and come back home. I don’t want to let her move around because she is a girl. [Interviewer: You fear that she might get assaulted? ] Yes.” The vulnerability of children with DD to abuse is thus a pressing source of concern, particularly if incidents of harm occur beyond the oversight of trusted adults, in schools or on the long journeys which children with DD may have to travel to reach schools which accommodate them.

#### 3b. Priority for Action: Safety Considerations in Infrastructure and Manpower Decisions

Stakeholders suggested that classroom modifications could be implemented to prevent injuries of children within inclusive schools: “Their classes should be neat, beautiful, and safe. The floors should be covered with mattresses and other comfortable materials to protect them from injury.” (A21, teacher). The necessity of enhanced supervision for children with DD was also highlighted: “The teachers should watch the children with special needs so no one should get hurt in the classroom.” (A35, teacher). Beyond classrooms, enhanced supervision can also alleviate concerns of children escaping and running away from schools.

Regarding the prevention of sexual abuse, stakeholders’ suggestions similarly included considerations of children’s safety incorporated into the school’s infrastructure design, alongside close supervision. For instance, a school principal mentioned building toilets for students in close proximity to staff: “They [other students] sometimes try to harass and rape the female students. We tried to solve this problem by building toilets closer to staff offices.” (A21, school principal). The importance of close observation was again highlighted in relation to cases where the child may be unable to report abuse:

(Interviewer: What could be done about it [sexual abuse]? ) Uh, well, close observation, and in this case you know, they don’t… if they get beaten or any sexual abuse or whatever, they can’t, you know, explain. They can’t tell you. So it’s very close observation. (E08, academic)

### Theme 4: Societal Inclusion of People with DD

#### 4a. Benefit: Greater Acceptance of People with DD in the Community

Inclusive education was seen as a way to create an inclusive society, responsive to the needs of people with DD, by raising all children to become welcoming members of the community, as well as by creating awareness of children with DD more generally in the society.

First, stakeholders reported that a mindset of acceptance towards diverse abilities can be nurtured among typically developing children who learn alongside children with DD:

Children who learn in inclusive education, they are positive towards this diversity, you know, they accept individual differences. […] They acknowledge the different potentials, they’re not like you know, children who only lack or some shortage, they acknowledge their potential. (E04, school representative)

Typically developing children who recognise the importance of inclusion can then act as advocates for their peers with DD, with NGO representative E09 describing student volunteers assisting with programmes to raise awareness about DD in their schools or the wider community. Furthermore, typically developing children may be motivated to continue supporting individuals with DD as they mature, even using their careers to do so:

They are inspired, they want to tell you about autism, ADHD, speech delay, the cause, the treatments. Some would say I want to be a neurosurgeon to help children who suffer with epilepsy. You know, when you learn, you think about that person. You don’t exclude [them] from your life, so it’s even inspiring them to study about more of this developmental issue. (E04, school representative)

Secondly, inclusive education highlights the capacity of children with DD to learn and socialise with typically developing peers, fostering inclusive attitudes in the community, beyond the boundaries of inclusive schools, starting from children’s families. Government representative A29 noted that among families of children with DD, “attitudes toward their children will change. They will start to think that their children are teachable and can become successful if they get proper support.” The potential for inclusive education to inspire positive changes in broader community attitudes towards children with DD was widely acknowledged:

It will make people aware of the condition, and the children will get support from the community. […] If people are aware of the condition, they will not stigmatise slowly developing children. They will not think of them as foolish but accept them as part of the community. (A34, teacher)

While including children with DD in inclusive schools may initially make existing stigma in the community surface, this could be seen by stakeholders as an opportunity to address negative beliefs. Sustained negative attitudes towards inclusive education were reported to be especially prevalent among caregivers of typically developing children, who opposed the notion of children with DD learning alongside their children. E04, a representative from an inclusive school, noted that such sentiments were expressed when the school’s inclusive education programme was initiated: “they said [children with DD] have their own place, why they come, why do they learn with us?” E04 takes this opportunity to have regular conversations with parents, making them aware of the ability of children with DD to learn and of their human right to be included.

Inclusive education was described as fostering a culture which accepts and embraces children with DD alongside other children. As the community grows to appreciate each child as a capable individual, they can also become more mindful of the unique needs of children with DD and recognise their responsibility to offer support, as “everyone is responsible, as citizens, to help and support special needs children” (A23, teacher).

#### 4b. Disadvantage: Poorly Implemented Inclusion Reinforces Stigma

On the other hand, inclusion in mainstream schools may have the opposite effect if children with DD are not supported adequately, or if inclusive programmes perpetuate segregation models.

The relationship between a lack of support for children with DD and stigma was latent in participants’ responses rather than fully articulated. However, it was clear that the challenges of poorly supported children with DD to assimilate into mainstream classrooms may exacerbate others’ intolerance of them. Especially when children struggle to communicate or display challenging, disruptive behaviours—which may be aggravated by hostility and neglect faced at times in inclusive schools—they may be rejected by schoolteachers and principals:

They cry, they shout, they roll in the ground and so on. So all these things makes the school or the teacher or the school principal not, you know, accommodating such kind of condition. (E12, clinician)

Some schools in Addis Ababa have implemented inclusive education in a broader sense, through the creation of special units within mainstream schools. This arrangement was also seen by a minority of stakeholders as perpetuating exclusion in the society:

They say it is inclusive, but practically if you visit them, they give them a different classroom as I told you, a kind of exclusion within inclusion. They say, they report that they are including them, but the practical process, education process in the school, I don’t think they’re included, inclusive processes are not happening. (E03, consultant)

The intervention does not understand what’s really happening, right now on the ground. So they think that it’s addressed, but in actual situation, it created a segregation. I must put it in that way. Created segregation. (E16, school representative)

Thus, inclusive education programmes that are poorly implemented could emphasise the differences of children with DD from other children and cast them only in terms of deficits, building hostility towards children with DD. Special units schemes used to implement inclusion with an appropriate level of support, while preferred by many stakeholders, according to others may paradoxically reinforce inclinations to exclude them from schools and the society.

#### 4c. Priority for Action: School Readiness for Children with DD

Stakeholders called for preparatory training in special units preceding inclusion in mainstream classes, following on from success stories of a few private and government inclusive schools and of children from special schools then included in mainstream classes.

Through these programmes, the primary challenges of children with DD which may elicit stigma can be addressed prior to their inclusion, making the integration process smoother: “when they come, you know, and you include them directly, children would be afraid of them, you know when they misbehave, do some inappropriate, you know, unusual behaviors, but now we deal with this before they enter to the regular programme” (E04, school representative).

Participant E04 also described how the use of school readiness classes makes the transition to inclusive classrooms easier for the child and reduces their displays of challenging behaviour:

After we engage them with these activities, they improve… with their behavior. They are familiar with the compound, they are familiar with the teachers, they are familiar with where the toilet is, with everything. So when we see them they are ready, we bring them to you know, the inclusive, for the regular program. (E04, school representative)

Based on similar positive examples, stakeholders argued that preparatory training for inclusion should involve toilet training, behavioural management, sensory integration, and basic learning skills:

They try to teach them basics…. How to dress, how to use toilet, how to communicate with people, the basics that we learn from the very beginning of childhood, if they learn the basics in schools and the others… they shall learn with other students without disabilities. (E03, consultant)

## Discussion

This analysis is reported as part of a broader exploratory study that aimed to understand the preparedness, context and processes for the implementation of inclusive education in Addis Ababa. In this in-depth analysis, we sought to understand whether stakeholders deem inclusive education to be a practice that can adequately support the needs of children with DD in this context, and how optimal outcomes can be achieved. Such consideration is crucial before any attempts to develop models for implementation, as we seek to do in Genovesi et al. (2023, 2024).

We explored stakeholders’ views on the ways in which inclusive education can support the needs or exacerbate the challenges of children with DD in Addis Ababa, Ethiopia. We also highlighted priorities for action to make inclusive education an acceptable way to address the needs of children with DD in this context. Our analysis showed that inclusive education can support the needs of children with DD in relation to their learning and development, social relationships, safety, and inclusion in society. At the same time, it can also exacerbate their challenges in all these domains. Stakeholders have highlighted potential positive and negative consequences of inclusive education based on their experiences. On one hand, children with DD can be supported to learn and participate alongside typically developing children, and forge positive relationships with peers in school, promoting their acceptance as valued members of the wider society. On the other hand, the learning difficulties of children with DD may be amplified in inclusive education, and children may experience isolation or even abuse from peers and teachers, perpetuating the exclusion of children with DD in society. These positive and negative consequences are not mutually exclusive, but may occur alongside one another; the findings highlight the importance of managing potential disadvantages of inclusive education so as to maximise its potential in meeting the needs of children with DD. Priorities for action include enhanced teacher training, awareness raising among the school community of the nature of DD, safety measures to prevent harm and abuse, and ensuring the needs of children with DD are appropriately met before and during inclusion in mainstream classes.

While existing quantitative research, mainly from Western countries, is currently inconclusive (Dalgaard et al., 2022), in the present study, participants expressed that inclusive education could benefit the development of children with DD in Ethiopia through enabling their collaborative learning alongside typically developing peers. Indeed, peer support had previously been reported across several African countries as a successful strategy to support learning for children with DD in inclusive classes (Genovesi et al., 2022). Similarly, in Western inclusive education practice, peer support is central to peer-mediated instruction, and evidence-based practice improving academic outcomes for autistic children (Haas et al., 2020). However, inclusive education may be unsatisfactorily implemented in low-resource contexts, leading to children with DD being overlooked and unable to progress in academic settings (Eleweke & Rodda, 2002), with similar concerns being expressed in the present study. A key factor contributing to undesirable outcomes is inadequate competency of teachers to educate children with disabilities effectively, a finding observed across developing countries such as India (Das et al., 2013), Zimbabwe (Shadreck, 2012) and various countries across Africa (Genovesi et al., 2022). Inadequate preparation for teachers to teach children with disabilities in inclusive settings has also been previously reported as a challenge in Ethiopia (Beyene & Tizazu, 2010; Franck & Joshi, 2017; Ginja & Chen, 2021). To ensure that inclusive education delivers optimal outcomes for the learning and development of children with DD, it is crucial to make appropriate training available to all relevant teachers. Stakeholders recommended that such training should be specific to DD, as current training efforts and literature generally consider inclusive education to cater to children with disabilities in general, which has led to a disproportionate focus on supporting children with sensory and physical disabilities compared to children with DD. Based on stakeholders recommendations, teacher training should also aim to develop specific competencies rather than teach general theoretical knowledge, in line with previous research reporting teachers’ lack of familiarity with specific teaching strategies for inclusive education (Ginja & Chen, 2021). Hands-on experience of supporting children with DD in classrooms was also considered to be essential. Lastly, stakeholders called for training to be repeated over time rather than being one-off in nature, as continual professional development is crucial to equip teachers with relevant skills and knowledge to perform in their roles as teachers (Berry et al., 2011).

Potential dangers to children’s safety were raised as key concerns of inclusive education. Notably, the risk of sexual abuse of children with DD is not unique to Ethiopia — children and adults with DD in the UK and USA are also at increased risk of experiencing sexual abuse compared to their typically developing peers (Byrne et al., 2017). Additionally, children with disabilities are generally at increased risk of abuse compared to the typically developing population (Legano et al., 2021) and may experience abuse in other educational settings. For example, a study in Botswana reported such abuse in special schools (Shumba & Abosi, 2011). However, stakeholders in our study often considered safety risks as a specific downside of inclusive schools. The vulnerability of children with DD to harm may be exacerbated in inclusive education settings due to the interactions between children with DD and typically developing peers, and the limited capacity of teachers to supervise students. In particular, the phenomenon of children with DD experiencing bullying by their typically developing peers is widespread. Compared to typically developing children and children with other types of disabilities, children with DD, including those with autism (Park et al., 2020) experience higher rates of verbal assaults and bullying victimisation (Glumbić & Žunić-Pavlović, 2010; Morrison, 1994). Park et al. also reported that inclusive school settings were related to higher levels of victimisation compared to segregated school settings. These findings reinforce the importance of bullying prevention interventions, as discussed in Theme 2, for inclusive education to be a safe environment experienced positively by all children. Conversely, suggestions to counter the issue of sexual abuse were limited in this study, predominantly based around close observation of children. As a systemic issue, it is likely that interventions to address this would be needed on a wider scale, addressing broader societal attitudes towards disability and gender, rather than being restricted to the educational sphere. However, more specific strategies, such as that suggested by our participant of building girls’ toilets closer to staff offices, may be beneficial in the short term as measures to protect children’s safety within inclusive schools. Additionally, several schools, including special schools in Addis Ababa, implement the safeguarding measure of prohibiting one teacher from being alone with one student at any time.

Finally, inclusive education can reduce stigma and enhance acceptance (Kart & Kart, 2021), therefore driving social inclusion, conceptualised as people with DD participating and forming interpersonal relationships in community settings (Simplican et al., 2015). However, instances of exclusion of children with DD can be preserved even with inclusive education (Woodgate et al., 2019). Children presenting with frequent behavioural challenges or high support needs in adaptive skills can be negatively perceived by the community, reinforcing negative beliefs. When these difficulties cannot be adequately addressed in inclusive settings, they persist, generating further stigma. As such, stakeholders suggested that such needs should first be addressed in special education settings. This is consistent with understandings of inclusion previously reported in other African countries, where inclusive education is seen as an environment for children who can “cope” with it and “adapt” (Bannink et al., 2019; Brydges & Mkandawire, 2018). Bannink et al. stressed that such understanding is not in contrast with a human rights framework. Indeed, the approach proposed by stakeholders in our study enables the higher-support needs of children with DD to be addressed more comprehensively, and it does not preclude their inclusion when their more severe difficulties have been addressed. Similarly, stakeholders may not necessarily reject full inclusion as an ultimate long-term goal, but they recognise current difficulties and the need for a stepwise approach, to avoid inadvertent harm that a sudden change to full inclusion could cause.

### Limitations and Further Research

A key limitation of the present study is that we did not gather perspectives from children with DD directly, nor of adults with DD. This is particularly pertinent to the research question of how their needs are supported or exacerbated in inclusive education, as we lack their first-hand perspectives on the issue. While arguments based on the rights of people with DD to be included in research that concerns them (e.g. Walmsley, 2001) are compelling alone, they are affirmed by the critical insights revealed through research involving people with DD, which may not otherwise be shown. For instance, supports intended to facilitate the inclusion of children with DD in education were reported by children to be undesirable when associated with dependency and stigmatisation (Woodgate et al., 2019). Our study does not involve children or adults with DD as participants or team members because people with DD in the Ethiopian context, especially those with milder difficulties, are often not identified in the community, or stigma and other contextual barriers prevent them from accessing services. As a result, most children diagnosed with DD in this context have moderate to profound forms of DD that severely impair communication, limiting their ability to engage in interviews or supported conversations. While efforts to promote meaningful involvement of people with DD in this context are ongoing, we have been unable to do so in the current study. We mitigate this limitation partially by including a caregiver of a child with DD in our team and interviewing ten caregivers of children with DD, who are keenly aware of their children’s needs and characteristics. However, they cannot speak perfectly on behalf of their children, who are autonomous individuals, thus future research to directly explore the sentiments of children with DD in Addis Ababa in relation to inclusive education can be conducted.

Additionally, our analysis focused on children’s outcomes, but it needs to be acknowledged that implementing inclusive education can have diverse effects and implications on other stakeholders. In turn, these are key factors influencing stakeholders’ commitment to implementing inclusive education. While children with DD are the primary target when establishing inclusive education in mainstream schools, outcomes and factors relevant to other key implementation agents, such as teachers, and recommendations for building enabling contexts for all are discussed elsewhere (Genovesi et al., 2023, 2024).

Further, as the study was conducted in Addis Ababa alone, the results may not be applicable to other settings within Ethiopia, Therefore, it may be useful for follow-up studies to be conducted in alternative settings, particularly in more rural and resource-poor areas than the capital city where most resources, especially those in relation to the education of children with special needs, are concentrated (Tekola et al., 2016). This is because additional barriers to inclusive education for children with DD may exist in such areas, and these would be important to identify as they should be addressed if inclusive education were to be incorporated into the education system on a country-wide scale.

## Conclusion

Inclusive education has the potential to both support the needs and exacerbate the challenges experienced by children with DD in Addis Ababa, Ethiopia, at the levels of children’s learning and development, social relationships, safety, and inclusion in society. Inclusive education, when appropriately implemented, can be a means to expand the limited access of children with DD to education and inclusion in the society. As such, we believe it is important to strive towards implementing inclusive education in Addis Ababa, while being mindful of potential drawbacks for children with DD and applying recommendations to manage them. Therefore, Genovesi et al. (2023, 2024) report an analysis of the study dataset that leads to a full framework-based overview of both relevant contextual factors (Genovesi et al., 2024) and an implementation model (Genovesi et al., 2023) for the inclusion of children with DD in primary schools. Building from local understandings of inclusive education and its potential benefits and disadvantages for children with DD, we can strive for an education system in which children with DD fully participate as students and members of the society alongside typically developing peers.
